# Depression in fathers in the postnatal period: Assessment of the Edinburgh Postnatal Depression Scale as a screening measure

**DOI:** 10.1016/j.jad.2010.01.069

**Published:** 2010-09

**Authors:** Olivia J.H. Edmondson, Lamprini Psychogiou, Haido Vlachos, Elena Netsi, Paul G. Ramchandani

**Affiliations:** a Section of Child and Adolescent Psychiatry, University of Oxford, Oxford, UK; b Milton Keynes Specialist CAMHS, Milton Keynes, UK

**Keywords:** Depression, Postnatal, Fathers

## Abstract

**Background:**

Postnatal depression commonly affects women after the birth of a child, and is associated with an increased risk of adverse outcomes for their children. A wide variety of measures have been used to screen for depression in the postnatal period but little research has investigated such measures with men. However depression can also affect men at this time, and this is associated with an independently increased risk of adverse child outcomes. The present study aimed to determine whether a reliable cut off point for the Edinburgh Postnatal Depression Scale (EPDS) can be established to screen fathers.

**Method:**

A sample of fathers was sent the EPDS at 7 weeks after the birth of their child. A structured clinical interview was conducted with 192 men to determine whether they were suffering from depression.

**Results:**

Fathers with depression scored significantly higher on the EPDS than non-depressed fathers. A score of greater than 10 was found to be the optimal cut off point for screening for depression, with a sensitivity of 89.5% and a specificity of 78.2%.

**Limitations:**

The relatively modest participation rate means the results may not be fully generalisable to the whole population.

**Conclusion:**

The EPDS is shown to have reasonable sensitivity and specificity at a cut off score of over 10. The study shows that it is possible to screen fathers for depression in the postnatal period and it may be valuable to administer this measure to new fathers.

## 1 Introduction

Depression is known to be a common disorder in women in the postnatal period with an estimated prevalence of 13% (O'Hara and Swain, 1996). Postnatal depression in mothers has been shown to impact negatively on their infant's cognitive, social and behavioural development (Murray, 1992). Research is beginning to suggest that depression can also affect fathers in the postnatal period, with incidences of between 1.2% and 25.5% in community samples (Goodman, 2004); although larger studies suggest prevalence rates at the lower end of this range (3–10%) (Ballard et al., 1994; Matthey et al., 2000; Ramchandani et al., 2005; Spector, 2006; Schumacher et al., 2008). Depression in fathers in the postnatal period may have an independent adverse effect on child development (Ramchandani et al., 2005; Ramchandani et al., 2008; Paulson et al., 2009). Kane and Garber (2004) have linked paternal depression to child psychopathology and a review by Phares et al. (2002) suggested an association between depression in fathers and a variety of negative emotional and behavioural outcomes in children. However, overall this area has received relatively little research attention and there are no programmes of routine screening of fathers for depression in the postnatal period.

A wide variety of measures have been used to screen women for depression in the postnatal period. These include the Beck Depression Inventory (Beck et al., 1961) and the Centre for Epidemiological Studies-Depression Scale (Radloff, 1977). However particular care needs to be taken in the postnatal period as some of the features of depression identified in these scales, such as sleep disturbance, changes in appetite and weight loss, are normal features of postpartum adaptation. One measure which largely avoids these biological symptoms of depression is the Edinburgh Postnatal Depression Scale (EPDS; Cox et al., 1987). The EPDS has been used widely in studies investigating postnatal depression in women, and has been used in some studies to assess depressive symptoms in men as well (Matthey et al., 2001; Ramchandani et al., 2005).

The majority of studies utilising the EPDS use a cut off score of greater than 12 (Murray and Carothers, 1990) (from a range of 0–30), although other cut off scores have been used (e.g. > 13; Murray and Cox, 1990). Studies using the EPDS with men have used a range of different cut off scores (> 12, Ramchandani et al., 2005; > 10, Madsen and Juhl, 2007; > 9, Areias et al., 1996). However, to date Matthey et al. (2001) have been the only authors to examine the utility of the EPDS in fathers, assessing its validity against a clinical assessment for depression. They concluded that the EPDS is a sensitive and specific screener for mood in fathers, and found that a score of > 9 was the optimum cut off for depression, decreasing to > 5 if caseness is widened to include anxiety disorders. If the EPDS is to be consistently used to screen for depression in new fathers it is important that a reliable cut off point is determined and established in different populations.

The aim of the present study was to ascertain the effectiveness of the EPDS as a screening tool in new fathers in the UK, by examining its validity against a structured clinical interview.

## 2 Method

### 2.1 Participants and procedure

Couples were recruited from the postnatal maternity wards of the John Radcliffe Hospital, Oxford and Milton Keynes General Hospital. They were invited to participate in the study by trained staff (all graduate psychologists) and provided with an information leaflet describing the study. Seven weeks after the birth of their child all consenting fathers were sent a questionnaire including the Edinburgh Postnatal Depression Scale (EPDS). Reminders were sent to those who did not reply within 2 weeks. Questionnaires were sent to 4107 fathers, 1562 (38.0%) were returned. We subsequently attempted to contact all fathers scoring 10 or above on the EPDS by telephone, and a 1 in 4 random sample of fathers with a score less than 10. Those agreeing to participate further were visited at home when their infant was approximately 14 weeks old. There was a mean time gap of 4.8 weeks between the return of the EPDS and the following home visit. During this visit each parent signed a consent form and was interviewed using the Structured Clinical Interview for DSM-IV (SCID — Depression and Anxiety Disorders Sections), without their partner being present. The interview was conducted by a psychologist or psychiatrist trained in administering the SCID. Participants were given further questionnaires to complete, including the EPDS for the mother. In total, 340 couples were invited to take part and 192 (56.6%) couples agreed to the home visit.

### 2.2 Measures

The Edinburgh Postnatal Depression Scale (Cox et al., 1987) consists of 10 self-report items. Each item is scored from 0 to 3, yielding a total range of 0–30. It has been found to have satisfactory sensitivity and specificity among women (Murray and Carothers, 1990) and is sensitive to changes in the severity of depression over time (Cox et al., 1987).

The Structured Clinical Interview for DSM-IV (First et al., 1997) is a semi-structured interview for making the major DSM-IV Axis I diagnoses. The SCID has been found to have high reliability (Zanarini et al., 2000) and validity (Basco et al., 2000).

### 2.3 Statistical analysis

Descriptive analyses of the demographic characteristics of the sample were undertaken (mean ages, marital status, infants' ages and child birth order). The proportion of fathers and mothers with a history of depression was also calculated.

The mean EPDS scores of the depressed and control group fathers were compared. Mann–Whitney test was used, as the EPDS scores were not normally distributed. Receiver operating characteristics were then calculated to determine the sensitivity, specificity and positive and negative predictive values (PPV and NPV) of each EPDS cut off score for fathers compared to the diagnosis of major depressive disorder in the structured clinical interview (SCID). As the sample had been preferentially selected for high EPDS scores (only 1 in 4 low scoring fathers were approached compared to all high scoring fathers), we subsequently recalculated the receiver operating characteristics for a modelled data set with 4 times the number of low scoring fathers to approximate the original sample population. Finally we calculated the sensitivity, specificity and positive predictive value (PPV) of each EPDS cut off point for mothers in the study.

## 3 Results

### 3.1 Participants

The overall sample size was 192. Complete data was available for 189 fathers and 184 mothers. The mean age of the men was 35 years (SD = 5.86) and the mean age of the women was 33.3 years (SD = 4.84). The mean age of the infant was 14.5 weeks (SD = 3.04). All but one set of parents were either married and/or living together as a couple. The majority (59.4%) were first time parents.

In total, 19 fathers met the criteria for depression (10%) and 12 (6.3%) met the criteria for Generalised Anxiety Disorder (GAD). There were 5 fathers who had both depression and GAD. Furthermore, 35 fathers (18.4%) had a history of depression.

When the mothers were interviewed, 7 (3.7%) met the criteria for depression. Forty-five (23.8%) had a history of depression.

### 3.2 Mean EPDS scores for caseness

Fathers with depression (diagnosed by clinical interview) scored significantly higher on the EPDS than non-depressed fathers (depressed group Mean score = 14.79, SD = 3.41; non-depressed Mean score = 6.64, SD = 4.40; *U* = 258.00, *p* < 0.001).

### 3.3 Receiver operating characteristics (ROC)

Table 1 shows the results from the ROC analysis. The resulting Area under the Curve was 0.916 (95% confidence interval: 0.864–0.967). A score of greater than 10 was selected as the optimal cut off point to screen for major depressive disorder in fathers, as a high level of sensitivity is required for a screening test for depression. At this cut off, 89.5% of the depressed fathers and 78.2% of the non-depressed fathers are classified correctly, giving an overall accuracy of 79.4% (Chi2 = 27.2; *p* < 0.01). When the analysis was rerun using the expanded database with more participants scoring low on the EPDS, the > 10 cut off yielded a sensitivity of 77.3% and specificity of 92.9%.

If the definition of caseness is broadened to include GAD, the optimum cut off is lowered to scores over 8. At this cut off 92.0% of the depressed and anxious fathers and 66.5% of the non-depressed/non-anxious fathers are classified correctly. Finally, for mothers in this sample, the optimum cut off for screening for depression is a score greater than 9. At this cut off 85.7% of the depressed mothers, and 83.6% of the non-depressed mothers are classified correctly.

## 4 Discussion

The results of this study suggest that the EPDS could be a useful tool for screening for depression in fathers. The optimum cut off score is > 10 to detect cases of major depressive disorder. The optimal cut off decreases to > 8 when the diagnosis of caseness is broadened to include GAD. The depression cut off point is comparable to that found by Matthey et al. (2001) in the only previous study to look at the utility of the EPDS in fathers. In their Australian study, they found an optimum cut off of > 9, though this decreased to > 5 if caseness included anxiety disorders. However they included minor depression in their analysis, as well as major depression, which may explain the slightly lower cut off point. It is of note that the cut off score for mothers for depression in the present study (> 9) is lower than that found in a number of other studies. This may be in part because of the low prevalence of depression in women in this sample (3.7%) which, in turn, is probably a result of the method of sample selection used, as this study focussed primarily on fathers.

The study has a number of strengths and limitations. Just under 200 fathers were recruited from general maternity services and interviewed in the perinatal period using a structured psychiatric interview. However the findings from this sample may not be entirely generalisable to the general population. This is in part because the response rate was modest (although higher than in some other studies addressing paternal depression), and also because fathers were preferentially selected for the SCID interview based on high EPDS scores. It was also found that older men were more likely to participate. There was a gap of 4.8 weeks between fathers returning the EPDS and the SCID being administered. It is possible that depressive symptoms may have changed over this period. However, there is evidence for satisfactory test–retest reliability of the EPDS in women (Guedeney and Fermanian, 1998) and scores in men show reasonable stability over time (Ramchandani et al., 2008). In addition, a recent systematic review found that the predictive validity of the EPDS in women was not affected significantly by a time gap of even greater than 8 weeks between administration of the EPDS and the clinical interview (Gibson et al., 2009). However, we do not know whether the same applies to fathers, and so future studies should aim to deliver the instruments at the same assessment.

Increasing evidence suggests that depression in fathers after the birth of the child is associated with an adverse impact on child development, independently of the mother's mood (Ramchandani and Psychogiou, 2009). This suggests that successful detection and treatment of depression in both mothers and fathers in the postnatal period could be critical for ensuring the best possible outcome for the child. The current study shows that the EPDS has reasonable sensitivity and specificity at a cut off score of greater than 10, in a UK sample of fathers. The EPDS has a long track record of being routinely administered to mothers in the postnatal period. Given the prevalence of paternal depression in the postnatal period, the EPDS may be a useful tool to screen fathers in order to identify important difficulties at this sensitive time.

## Role of funding source

Funding for this study was provided by the Wellcome Trust (Grant 078434); the Wellcome Trust had no further role in study design; in the collection, analysis and interpretation of data; in the writing of the report; and in the decision to submit the paper for publication.

## Conflict of interest

The authors have no conflict of interest to declare.

## Figures and Tables

**Table 1:** Receiver operating characteristics using depression as the criterion (fathers).

EPDS cut off	Sensitivity	Specificity	PPV	NPV	Accuracy
> 6	100	52.9	19.2	100	57.7
> 7	100	60.0	21.8	100	64.0
> 8	100	65.3	24.4	100	68.8
> 9	94.7	68.2	25.0	99.1	70.9
> 10	89.5	78.2	31.5	98.5	79.4
> 11	78.9	84.7	36.6	97.3	84.1
> 12	68.4	90.6	44.9	96.2	88.3
> 13	63.2	94.1	54.5	95.8	91.0
> 14	52.6	96.5	62.5	94.8	94.8

*N* = 189; 19 with depression. PPV: Positive predictive value. NPV: Negative predictive value.
